# Hybrid Thoracoabdominal Aortic Aneurysm Repair After Prior Abdominal Aortic Aneurysm Repair: Safety and Outcomes

**DOI:** 10.1093/icvts/ivaf230

**Published:** 2025-09-27

**Authors:** Kazuhiro Ueno, Takashi Shuto, Takayuki Kawashima, Tomoyuki Wada, Katsuki Oji, Takeaki Dotsu, Norio Hongo, Yoshiki Asayama, Shinji Miyamoto

**Affiliations:** Department of Cardiovascular Surgery, Faculty of Medicine, Oita University, Oita, 879-5593, Japan; Department of Cardiovascular Surgery, Faculty of Medicine, Oita University, Oita, 879-5593, Japan; Department of Cardiovascular Surgery, Faculty of Medicine, Oita University, Oita, 879-5593, Japan; Department of Cardiovascular Surgery, Faculty of Medicine, Oita University, Oita, 879-5593, Japan; Department of Radiology, Faculty of Medicine, Oita University, Oita, 879-5593, Japan; Department of Radiology, Faculty of Medicine, Oita University, Oita, 879-5593, Japan; Department of Radiology, Faculty of Medicine, Oita University, Oita, 879-5593, Japan; Department of Radiology, Faculty of Medicine, Oita University, Oita, 879-5593, Japan; Department of Cardiovascular Surgery, Faculty of Medicine, Oita University, Oita, 879-5593, Japan

**Keywords:** hybrid repair, thoracoabdominal aortic aneurysm, abdominal aortic aneurysm repair, endovascular aortic aneurysm repair

## Abstract

**Objectives:**

Hybrid repair of thoracoabdominal aortic aneurysms (TAAA), combining visceral debranching and thoracic endovascular aortic repair, is a less invasive alternative to open surgery. However, data on long-term outcomes, especially in patients with prior open abdominal aortic aneurysm (AAA) repair, are limited. This study compares outcomes of hybrid TAAA repair in patients undergoing concomitant AAA repair vs those with prior open AAA repair.

**Methods:**

Between January 2007 and January 2024, 132 TAAA repairs were performed at our institution. We retrospectively analysed 80 patients who underwent hybrid TAAA repair. After excluding emergency cases and those without AAA repair, 67 patients were included: 50 with concomitant AAA repair (Group C) and 17 with prior open AAA repair (Group P). Perioperative outcomes, complications, and long-term survival and aortic event-free rates were compared between groups.

**Results:**

The median age was 72.0 years (IQR: 65.0-80.8) in Group C and 75.0 years (IQR: 70.0-82.0) in Group P (*P *= .34). Hospital mortality was 3.0% overall, with no significant group differences. Mean follow-up was 5.0 ± 3.1 years. Five-year overall survival was 69% in Group C and 63% in Group P (*P *= .22). Freedom from aortic events at 5 years was 92% in Group C and 83% in Group P (*P *= .19). IPTW-adjusted analyses confirmed no significant differences between the groups.

**Conclusions:**

Hybrid TAAA repair can be safely performed for patients with prior AAA open repair, with acceptable long-term outcomes after appropriate patient selection.

## Graphical abstract

**Figure:**
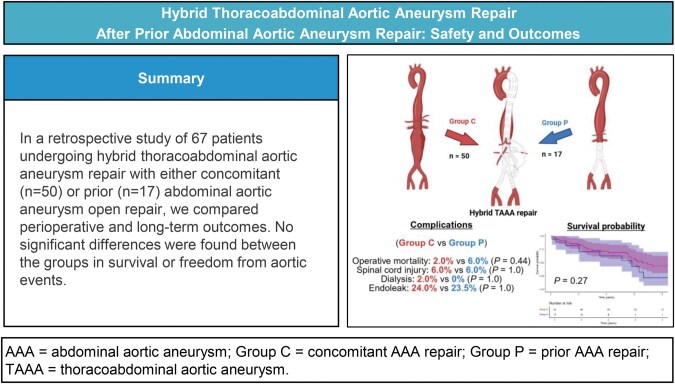

## INTRODUCTION

Thoracoabdominal aortic aneurysms (TAAAs) represent a complex and life-threatening condition requiring meticulous surgical intervention to prevent catastrophic outcomes such as rupture or dissection. Traditional open repair, while effective, is associated with significant morbidity and mortality due to the extensive surgical exposure and physiological stress imposed by a large lateral incision.^1^^,^^2^ In recent years, hybrid approaches combining abdominal aortic aneurysm (AAA) repair, visceral debranching procedure, and thoracic endovascular aortic repair (TEVAR) have emerged as a less invasive alternative, offering the potential for reduced perioperative complications and faster recovery in selected patients.^3^^,^^4^ However, with the increasing adoption of fenestrated and branched endovascular techniques (FB-EVAR) in many centres,^5–7^ hybrid TAAA repair is currently reserved for cases not amenable to FB-EVAR or open surgery.^8^^,^^9^ Despite this, there remains a paucity of data regarding its safety and long-term outcomes, particularly in patients with a history of prior AAA open repair. Concerns persist regarding the feasibility of hybrid TAAA repair in patients with prior AAA repair, as adhesions and altered anatomy may complicate the procedure.

The present study aims to address this gap by comparing the short-term and long-term outcomes of hybrid TAAA repair in 2 distinct patient populations: those undergoing a visceral debranching procedure with concomitant AAA repair and those with a history of prior AAA open repair. By evaluating the safety and efficacy of hybrid TAAA repair in these cohorts, this research seeks to provide valuable insights into the optimal management of TAAAs in patients with complex surgical histories. These findings may help inform clinical decision-making for this challenging patient population.

## PATIENTS AND METHODS

### Ethics statement

The retrospective study was approved by the Institutional Review Board of the Oita University Hospital (No. 2955; 24 October 2024), and the need for individual patient consent was waived. In accordance with the WMA Declaration of Taipei, the collection and storage of clinical data for future research use were approved by the same ethics committee, which also oversees ongoing data governance and biobank use.

### Data source

**Figure 1.**
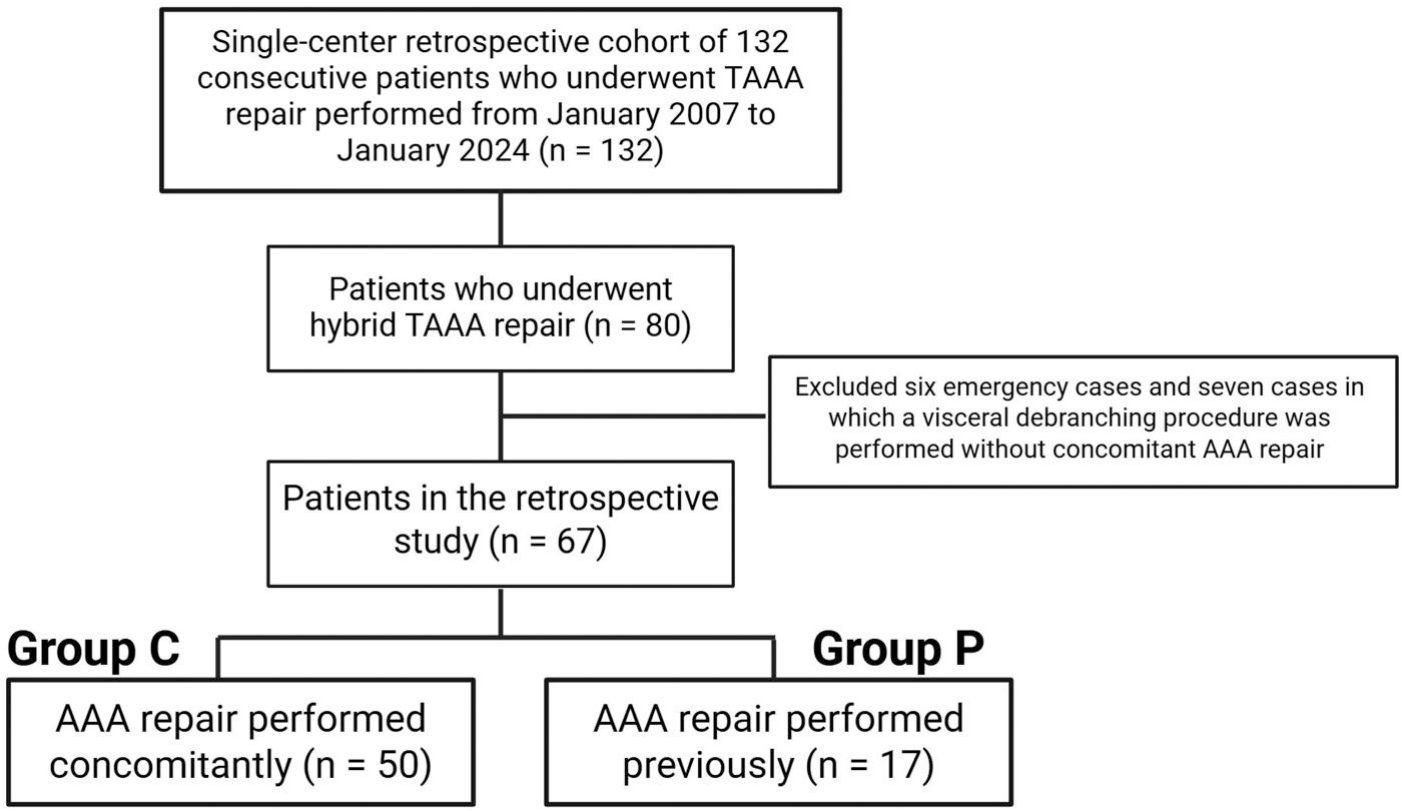
Study Design Overview. This study was designed as a single-centre retrospective analysis of 132 consecutive patients who underwent TAAA repair performed from January 2007 to January 2024. Among the 132 cases, 80 patients who underwent hybrid TAAA repair were evaluated. Six emergency cases and 7 cases in which a visceral debranching procedure was performed without concomitant AAA repair were excluded. Group C (*n* = 50) is the group in which abdominal aortic aneurysm repair was performed concomitantly, and Group P (*n* = 17) is the group in which abdominal aortic aneurysm repair was performed previously. Abbreviations: AAA: abdominal aortic aneurysm; TAAA: thoracoabdominal aortic aneurysm

This study was a single-centre retrospective analysis of 132 consecutive patients who underwent TAAA repair performed from January 2007 to January 2024. Among the 132 cases, we evaluated 80 patients who underwent hybrid TAAA repair. We excluded 6 emergency cases and 7 cases in which a visceral debranching procedure was performed without concomitant AAA repair. We compared the outcomes of 50 cases where AAA repair was performed concomitantly with a visceral debranching procedure (Group C) and 17 cases where AAA repair was performed previously (Group P), as shown in **Figure 1**. In Group P, all patients had previously undergone infrarenal abdominal aortic replacement with a Y-graft configuration. At our institution, hybrid therapy was initially applied between 2007 and 2011 to patients over 70 years to reduce surgical invasiveness, and from 2012 onwards, it was expanded to those over 60 with adequate reserve or comorbidities precluding open repair. Definitions of comorbidities, classification systems, and operative variables are provided in **Supplementary Methods**.

### Operative management

Our fundamental strategy for repairing TAAAs has been previously documented.^10^ Briefly, we perform the visceral debranching procedure and TEVAR in 2 stages for TAAAs. No patients underwent the debranching and TEVAR in a single-stage (same-day) setting; however, in selected patients with large aneurysms, TEVAR was performed on the following day after visceral debranching. The detailed operative strategy, graft configurations, and institutional changes in perioperative management are described in **Supplementary Methods**.

### Operative technique

**Figure 2.**
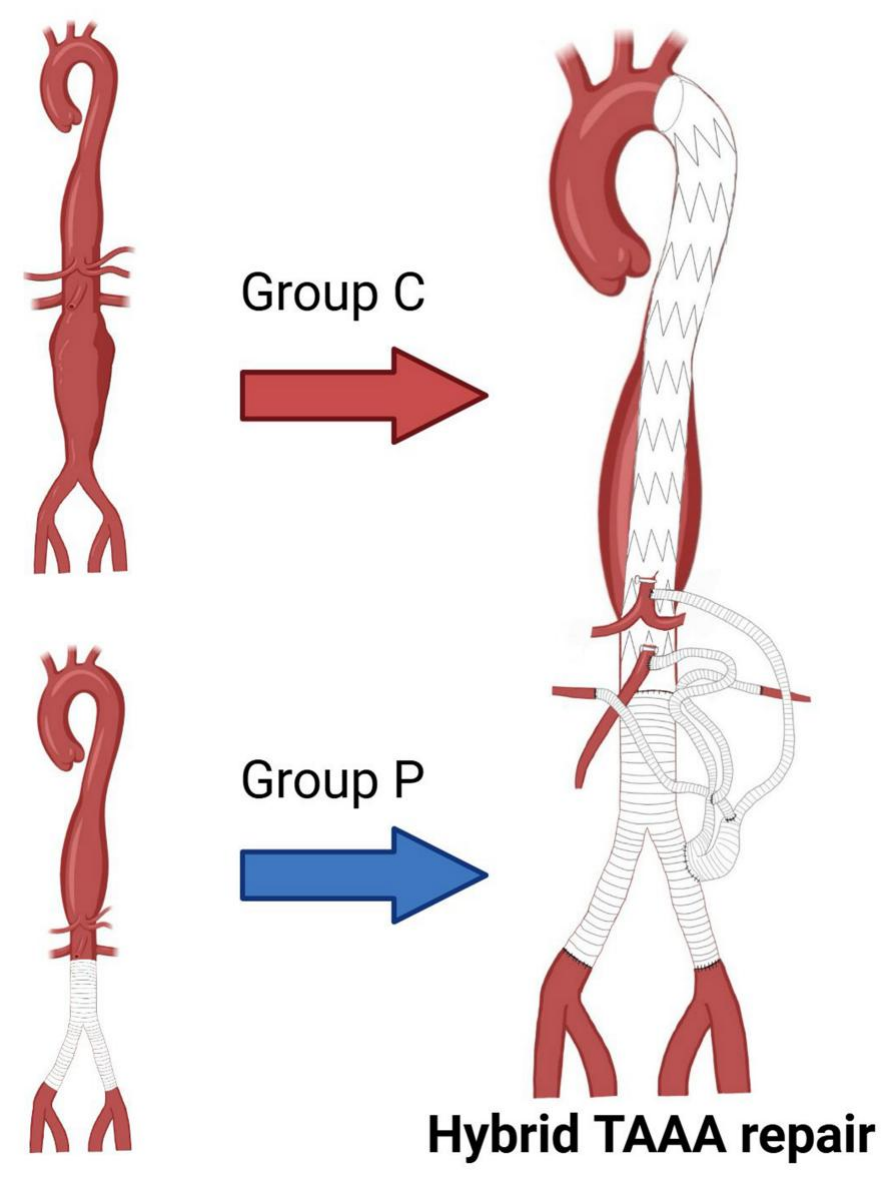
Quadrifurcated Visceral Debranching Graft Configuration in Hybrid TAAA Repair. Schema illustrating the quadrifurcated visceral debranching graft configuration applied in hybrid TAAA repair. Group C patients underwent concomitant abdominal aortic replacement with visceral debranching, whereas Group P patients had prior open AAA repair with subsequent visceral debranching. In both groups, the quadrifurcated graft received inflow from the pre-existing or concomitant abdominal prosthesis. Abbreviations: AAA = abdominal aortic aneurysm; TAAA = thoracoabdominal aortic aneurysm; TEVAR = thoracic endovascular aortic repair

Our methodology for the visceral debranching procedure has been previously documented,^10^ and TEVAR was conducted as a staged procedure. In patients with prior AAA repair, the visceral graft was connected to the existing Y-graft. **Figure 2** shows the quadrifurcated visceral debranching graft used in this series. Initially, the inflow anastomosis was anastomosed to a limb of the Y-graft. However, due to concerns about inadequate flow leading to postoperative renal dysfunction, the inflow was changed from approximately 2017 onwards to the graft joint between the trunk and limbs. This modification was described in detail in our previous institutional report.^11^ The term “graft limb” refers to one limb of the bifurcated Y-graft, while the ‘graft joint’ indicates the division point between the trunk and limbs. TEVAR was performed under local anaesthesia whenever possible. Detailed surgical techniques are described in **Supplementary Methods**.

### Postoperative follow-up

We evaluated all the cases with contrast CT at 1, 6, and 12 months and every year thereafter.

### Statistical methods

R Statistical Software (version 4.4.1) was used for all analyses. Continuous variables were compared using parametric or non-parametric tests as appropriate, and categorical variables were analysed using Fisher’s exact test. Kaplan-Meier analysis with log-rank testing compared survival and aortic event-free rates. Inverse probability of treatment weighting (IPTW) was used for adjustment; the sample size calculation, statistical assumptions, and detailed IPTW methodology are described in **Supplementary Methods**. Statistical significance was set at *P *< .05.

## RESULTS

### Patient characteristics

The patient characteristics are shown in **Table 1**. The study included 67 patients, with 50 patients in Group C and 17 patients in Group P. The median age of the cohort was 73.0 (IQR, 65.0-81.0 years), with no significant difference between Group C and Group P (72.0 years [IQR, 65.0-80.8] vs 75.0 years [IQR, 70.0-82.0], *P *= .34). All evaluated comorbidities showed no significant differences between the 2 groups (all *P *> .05). There were no significant differences in the distribution of the Crawford classification between Group C and Group P (*P *= .07). In Group P, the mean interval between the initial AAA repair and the visceral debranching procedure was 10.4 ± 4.2 years.

**Table 1.** Baseline Patient Characteristics in Concomitant (Group C) Vs Prior AAA Repair Groups (Group P) Undergoing Hybrid TAAA Repair

	All (*n *= 67)	Group C (*n *= 50)	Group P (*n *= 17)	*P*-value
Demographics				
Male gender	50 (74.6)	37 (74.0)	13 (76.5)	1.0
Age (years)	73.0 (65.0-81.0)	72.0 (65.0-80.8)	75.0 (70.0–82.0)	.34
Risk factors				
Hypertension	60 (89.6)	45 (90.0)	15 (88.2)	1.0
Diabetes mellitus	11 (16.4)	9 (18.0)	2 (11.8)	.72
Hyperlipidaemia	30 (44.8)	22 (44.0)	8 (47.1)	1.0
Renal insufficiency	46 (68.7)	33 (66.0)	13 (76.5)	.55
COPD	25 (37.3)	18 (36.0)	7 (41.2)	.78
Coronary artery disease	23 (34.3)	16 (32.0)	7 (41.2)	.56
Cerebral artery disease	9 (13.4)	5 (10.0)	4 (23.5)	.22
Aetiology				.67
Degenerative	46 (68.7)	33 (66.0)	13 (76.5)	
Dissecting	20 (29.9)	16 (32.0)	4 (23.5)	
Infectious	1 (1.5)	1 (2.0)	0 (0)	
Crawford classification				.07
Type I	9 (13.4)	8 (16.0)	1 (5.9)	
Type Ⅱ	14 (20.9)	12 (24.0)	2 (11.8)	
Type III	23 (34.3)	18 (36.0)	5 (29.4)	
Type IV	9 (13.4)	7 (14.0)	2 (11.8)	
Type Ⅴ	12 (17.9)	5 (10.0)	7 (41.2)	
Interval from AAA repair to visceral debranching procedure—years	10.4 ± 4.2	NA	10.4 ± 4.2	NA

Data are presented as *n* (%), mean ± SD, or median (IQR).

Abbreviations: AAA: abdominal aortic aneurysm; COPD: chronic obstructive pulmonary disease; NA: not applicable; TAAA: thoracoabdominal aortic aneurysm; TEVAR: aortic repair.

### Intraoperative details of visceral debranching procedure

Intraoperative details of the visceral debranching procedure are shown in **Table 2**. The mean operative time for the entire cohort was 521.0 ± 114.2 min, with no significant difference between Group C and Group P (515.6 ± 109.1 min vs 537.0 ± 130.1 min, *P *= .51). The median intraoperative blood loss was 2020.0 mL (IQR, 1325.0-2870.0), with no significant difference between Group C and Group P (2045.0 mL [IQR, 1372.0-2810.0] vs 2020.0 mL [IQR, 1120.0-4150.0], *P *= .58). The median number of anastomoses was significantly higher in Group C compared to Group P (10.0 [IQR, 8.0-10.0] vs 7.0 [IQR, 6.0-7.0], *P *< .001). The median number of reconstructed visceral arteries per patient was 4.0 (IQR 4.0-4.0) in the overall cohort, with no significant difference between Group C and Group P (4.0 [IQR 4.0-4.0] vs 4.0 [IQR 3.0-4.0], *P *= .18). There was also a statistically significant difference in the inflow source distribution between the groups (*P *= .005), with Group C having a higher proportion of left graft limbs (74.0%) compared to Group P (47.1%).

**Table 2.** Intraoperative Details of Visceral Debranching Procedure in Concomitant (Group C) Vs Prior AAA Repair Groups (Group P) Undergoing Hybrid TAAA Repair

	All (*n *= 67)	Group C (*n *= 50)	Group P (*n *= 17)	*P*-value
Operation time (min)	521.0 ± 114.2	515.6 ± 109.1	537.0 ± 130.1	.51
Bleeding (mL)	2020.0 (1325.0-2870.0)	2045.0 (1372.0-2810.0)	2020.0 (1120.0-4150.0)	.58
Number of anastomoses	9.0 (8.0-10.0)	10.0 (8.0-10.0)	7.0 (6.0-7.0)	<.001
Number of visceral anastomoses	4.0 (4.0-4.0)	4.0 (4.0-4.0)	4.0 (3.0-4.0)	.18
Inflow source				.005
Graft limb (left)	45 (67.2)	37 (74.0)	8 (47.1)	
Graft limb (right)	6 (9.0)	1 (2.0)	5 (29.4)	
Graft body	1 (1.5)	0 (0%)	1 (6.0)	
Graft joint	16 (23.9%)	12 (24.0)	4 (23.5)	

Data are presented as *n* (%), mean ± SD, or median (IQR).

Abbreviations: AAA: abdominal aortic aneurysm; TAAA: thoracoabdominal aortic aneurysm.

### Postoperative details and complications of visceral debranching procedure

The postoperative details and complications of the visceral debranching procedure are shown in **Table 3**. The median intensive care unit (ICU) length of stay was 2.0 days, with no significant difference between the 2 groups (*P *= .24). Complications were observed in a subset of patients, with no significant differences between Group C and Group P. There was no gastrointestinal injury in Group C or Group P (*P *= 1.0). No cases of bowel ischaemia were observed in either group (*P *= 1.0). The bleeding of the anastomosis site was observed in 6.0% of patients, with a non-significant rate in Group C compared to Group P (4.0% vs 11.8%, *P *= .26). Other complications (ileus, cholangitis, incisional hernia, and small bowel haemorrhage) were reported in 6.0% of patients, with no difference between the 2 groups (*P *= 1.0). Of 254 reconstructed branch grafts, follow-up imaging was available for 234 (92.1%). During a median follow-up of 1.8 years (IQR, 0.5-5.0), overall patency was 97.4%, with 6 occlusions (4 renal and 2 coeliac artery grafts). There was no significant difference between Group C and Group P (*P *= 1.0). All occlusions were asymptomatic and required no reintervention. An additional exploratory analysis was performed to assess the association between the inflow anastomosis site and graft patency. Although all 6 occluded grafts occurred in patients with non-graft joint inflow configurations, no statistically significant difference was observed in occlusion rates when compared to graft joint inflow (*P *= .59, **Table S1**).

**Table 3.** Postoperative Details and Complications of Visceral Debranching Procedure in Concomitant (Group C) Vs Prior AAA Repair Groups (Group P) Undergoing Hybrid TAAA Repair

	All (*n *= 67)	Group C (*n *= 50)	Group P (*n *= 17)	*P*-value
ICU length of stay (days)	2.0 (2.0-4.0)	2.0 (2.0-4.0)	2.0 (2.0-3.0)	.24
Complications				
Gastrointestinal injury	0 (0)	0 (0)	0 (0)	1.0
Bowel ischaemia	0 (0)	0 (0)	0 (0)	1.0
Temporary dialysis	6 (9.0)	4 (8.0)	2 (11.8)	.64
Permanent dialysis	1 (1.5)	1 (2.0)	0 (0)	1.0
Sepsis	2 (3.0)	2 (4.0)	0 (0)	1.0
Bleeding of anastomosis site	4 (6.0)	2 (4.0)	2 (11.8)	.26
Branch graft occlusion (per reconstructed branched)	6/234 (2.6)	5/184 (2.7)	1/50 (2.0)	1.0
Others	4 (6.0)	3 (6.0)	1 (6.0)	1.0

Data are presented as *n* (%) or median (IQR).

Abbreviations: AAA: abdominal aortic aneurysm; ICU: intensive care unit; TAAA: thoracoabdominal aortic aneurysm.

### Operative details and complications of TEVAR

The operative details and complications of TEVAR are shown in **Table 4**. The median operative time was 140.0 min (IQR, 100.0-183.0), with no significant difference between Group C and Group P (125.0 min [IQR, 99.3-167.3] vs 154.0 min [IQR, 141.0-210.0], *P *= .08). The median contrast medium volume was 88.5 mL (IQR, 60.0-110.0), with no significant difference between Group C and Group P (82.0 mL [IQR, 60.0-115.0] vs 100.0 mL [IQR, 73.0-105.0], *P *= .79). The median radiation exposure time was 31.6 min (IQR, 20.2-41.9), with no significant difference between Group C and Group P (30.8 min [IQR, 19.5-37.9] vs 34.6 min [IQR, 25.9-43.3], *P *= .30). The occurrence of SCI was 6.0% overall, with no significant difference between Group C and Group P (6.0% vs 6.0%, *P *= 1.0). Spinal cord injury (SCI) occurred in 4 patients: 3 cases of paraplegia and a case of paraparesis. SCI incidence was significantly higher in early TEVAR (<3 days) than in late TEVAR (≥4 days) (25% vs 1.8%, *P *= .016), with no significant difference between treatment groups (*P *= 1.0). Detailed case information is provided in **Table S2**.

**Table 4.** Operative Details and Complications of TEVAR in Concomitant (Group C) Vs Prior AAA Repair Groups (Group P) Undergoing Hybrid TAAA Repair

	All (*n *= 67)	Group C (*n *= 50)	Group P (*n *= 17)	*P*-value
Interval from visceral debranching procedure to TEVAR (days)	33.0 (7.0-87.0)	37.5 (7.0-94.5)	21.0 (7.0-63.0)	.39
Operation time (min)	140.0 (100.0-183.0)	125.0 (99.3-167.3)	154.0 (141.0-210.0)	.08
Bleeding (mL)	150.0 (50.0-300.0)	110.0 (50.0-290.0)	180.0 (50.0-400.0)	.47
Contrast medium volume (mL)	88.5 (60.0-110.0)	82.0 (60.0-115.0)	100.0 (73.0-105.0)	.79
Radiation exposure time (min)	31.6 (20.2-41.9)	30.8 (19.5-37.9)	34.6 (25.9-43.3)	.30
Material of stent graft				.59
Dacron only	53 (79.1)	42 (84.0)	13 (76.5)	
ePTFE only	13 (19.4)	7 (14.0)	4 (23.5)	
Dacron and ePTFE	1 (1.5)	1 (2.0)	0 (0)	
Hospital mortality	2 (3.0)	1 (2.0)	1 (6.0)	.44
Spinal cord injury	4 (6.0)	3 (6.0)	1 (6.0)	1.0
In early TEVAR (≤3 days, *n* = 12)	3 (25.0)	2 (22.2)	1 (33.3)	1.0
In late TEVAR (≥4 days, *n* = 55)	1 (1.8)	1 (2.4%)	0 (0)	1.0
Endoleak during the follow-up period	17 (25.4)	14 (28.0)	3 (17.6)	.53
Endoleak classification				
I	2 (10.5)	1 (6.3)	1 (33.0)	.30
Ⅱ	10 (52.6)	9 (56.3)	1 (33.0)	.58
III	5 (29.4)	5 (31.3)	0 (0)	.53
Unknown	2 (10.5)	1 (6.3)	1 (33.0)	.30

Data are presented as *n* (%) or median (IQR).

Abbreviations: AAA: abdominal aortic aneurysm; ePTFE: expanded polytetrafluoroethylene; TEVAR: thoracic endovascular aortic repair. *P *= .016 for early vs late spinal cord injury rate in the overall cohort.

### Long-term outcome of hybrid TAAA repair

**Figure 3.**
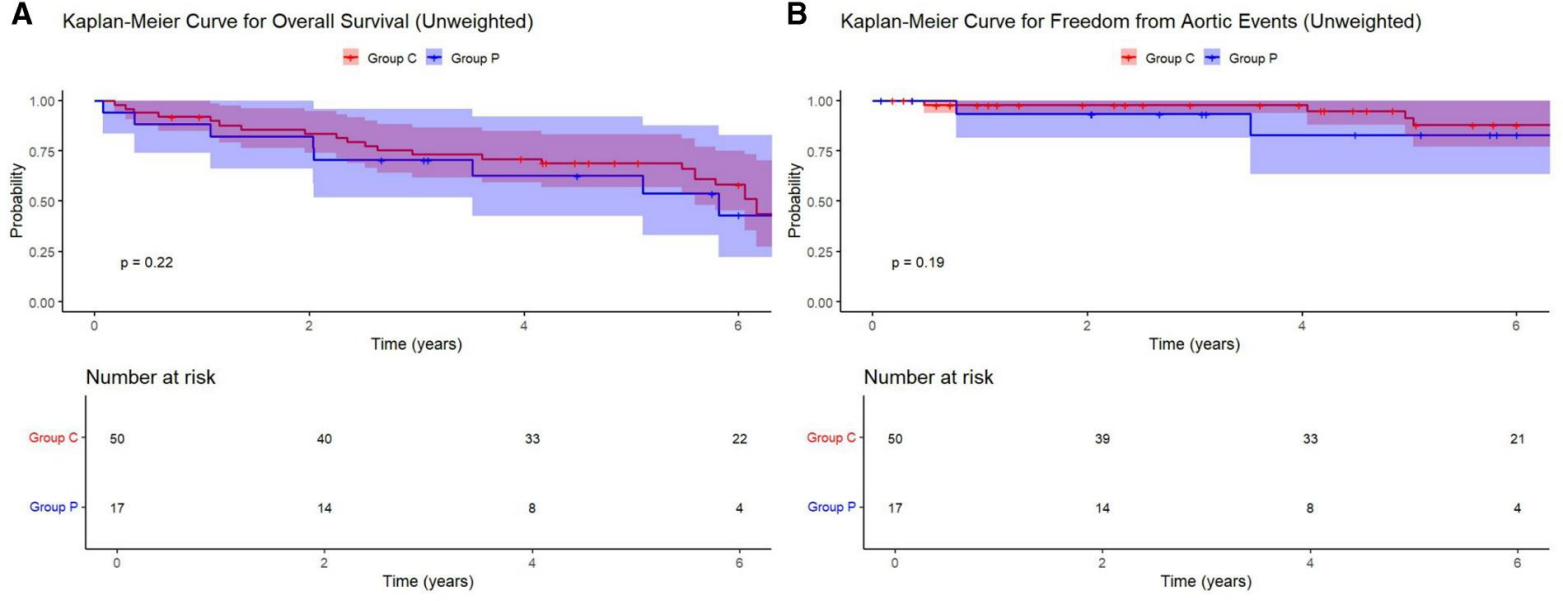
Comparison of Outcomes after Hybrid TAAA Repair between Patients With Concomitant Vs Prior Open AAA Repair. The mean follow-up period was 5.0 ± 3.1 years. Group C (*n* = 50) is the group in which abdominal aortic aneurysm repair was performed concomitantly, and Group P (*n* = 17) is the group in which abdominal aortic aneurysm repair was performed previously. (A) Overall survival. In Group C, overall survival was 84% at 2 years (95% CI, 74%-95%) and 69% at 5 years (95% CI, 57%-83%), compared to 82% at 2 years (95% CI, 66%-100%) and 63% at 5 years (95% CI, 43%-92%) in Group P (*P* = .22). (B) Freedom from aortic events. Freedom from aortic events was 98% at 2 years (95% CI: 94%-100%) and 92% at 5 years (95% CI, 82%-100%) in Group C, vs 93% at 2 years (95% CI, 82%-100%) and 83% at 5 years (95% CI, 64%-100%) in Group P (*P* = .19). These findings suggest comparable survival and procedural durability between the 2 groups. Abbreviations: AAA: abdominal aortic aneurysm; TAAA = thoracoabdominal aortic aneurysm

No patients were lost to follow-up during the study. Of note, there were 12 patients who underwent visceral debranching but did not proceed to TEVAR within the study period due to various clinical reasons (as detailed in **Table S3**). These patients were excluded from the present outcome analysis. Most of these patients underwent preventive visceral debranching at the time of AAA repair and had no immediate indication for second-stage TEVAR. The mean follow-up period was 5.0 ± 3.1 years. Endoleaks were observed during the follow-up period in 25.4% of patients (**Table 4**), with no significant difference between Group C and Group P (28.0% vs 17.6%, *P *= .53). There were no statistically significant differences in the distribution of endoleak types between the groups. **Figure 3A** shows the overall survival probabilities in each group. In Group C, the overall survival probability was 84% at 2 years (95% CI, 74%-95%) and 69% at 5 years (95% CI, 57%-83%) after hybrid TAAA repair, compared to 82% at 2 years (95% CI, 66%-100%) and 63% at 5 years (95% CI, 43%-92%) in Group P (*P *= .22). **Figure 3B** shows freedom from aortic events in each group. In Group C, the freedom from aortic events was 98% at 2 years (95% CI, 94%-100%) and 92% at 5 years (95% CI, 82%-100%) after hybrid TAAA repair, compared to 93% at 2 years (95% CI, 82%-100%) and 83% at 5 years (95% CI, 64%-100%) in Group P (*P *= .19). Several aortic events occurred during follow-up, including acute dissections, arch aneurysm enlargement, endoleaks, and graft infections. Their management is summarized in **Table S4**.

In an exploratory analysis using IPTW, no significant differences were observed in overall survival or freedom from aortic events between the 2 groups after adjustment. The IPTW-adjusted Kaplan-Meier curves are provided in **Figures S1**  **and**  **S2**. After IPTW adjustment, the balance of baseline characteristics between groups was confirmed with standardized mean differences below 0.2 for all variables (**Table S5**).

## DISCUSSION

Our findings suggest that hybrid TAAA repair can be safely performed in patients with a history of prior AAA open repair, with comparable short- and long-term outcomes to those undergoing concomitant AAA repair.

In this study, the baseline characteristics of Group C and Group P were generally well matched. Although the median age was slightly younger in Group C compared to Group P, this difference was not statistically significant, suggesting that age was not a major confounding factor in the comparison of outcomes.

Although it was expected that operative time would be prolonged during visceral debranching in patients with prior open AAA repair due to adhesions, in most cases, dissection was not particularly challenging. Typically, only the left or right limb of the previous graft needed to be dissected, and the time required for initial AAA repair could be omitted. As a result, the operative time was not significantly longer compared to cases where visceral debranching and AAA repair were performed concomitantly. Regarding intraoperative bleeding, similar to operative time, blood loss did not significantly increase during the abdominal debranching procedure in previously treated patients compared to concomitant AAA repair. This can be attributed to the reduced number of anastomoses because Group P did not require anastomoses involved in AAA repair (eg, central anastomosis, anastomoses to the iliac arteries). In terms of the inflow site, the left graft limb was mainly used, but in some cases, the right limb had to be chosen depending on the position of the limb and the state of adhesions, especially in Group P.

Short-term outcomes after the visceral debranching procedure were favourable, with similar ICU stays and complication rates in both groups. Importantly, prior AAA repair did not appear to increase the risk of anastomotic bleeding or technical difficulties, and no gastrointestinal injuries were observed. These results indicate that previous graft implantation does not adversely affect the safety or complexity of the visceral reconstruction.

Intraoperative parameters during TEVAR, including operative time, blood loss, contrast use, and radiation exposure, were comparable between groups, indicating that prior visceral debranching does not compromise the technical feasibility or procedural efficiency of the endovascular stage. Similarly, there was no difference in the choice of stent graft materials.

Hospital mortality and SCI did not differ significantly between Group C and Group P. Although prior lumbar artery coverage in Group P was expected to reduce SCI via collateral remodelling,^12^ no such advantage was observed in this cohort. Interestingly, when evaluating the entire cohort by TEVAR timing, SCI was significantly more frequent in patients undergoing TEVAR within 72 hours of debranching than after 4 days, consistent with previous reports suggesting insufficient collateral network maturation with shorter intervals.^13^ However, within early or late subgroups, Group C and Group P still showed no significant difference in SCI incidence. The theoretical advantage of staged repair for spinal cord protection, by avoiding simultaneous extensive spinal cord perfusion loss,^14^^,^^15^ was not clearly demonstrated in patients who had prior AAA open repair and subsequent hybrid TAAA repair. In 3 cases, SCI was associated with hypotension due to shock or sedation. These findings highlight the importance of stable haemodynamics, early limb assessment, and strict blood pressure control. At our institution, we aim to perform TEVAR under local anaesthesia whenever feasible, allowing direct postoperative neurologic monitoring, as described previously.^10^

Endoleaks remain a significant challenge following hybrid TAAA repair.^10^^,^^16^ In this study, the overall endoleak rate was relatively high (23.9%), but no statistically significant difference was observed between Group C and Group P. This suggests that endoleak development is more likely influenced by anatomical complexity or procedural factors during TEVAR than by the history of AAA open repair.

Although FB-EVAR is increasingly used in Western countries,^5^ its adoption remains limited elsewhere due to a lack of device approval or reimbursement. Since 2010, we have performed physician-modified FB-EVAR in selected patients, and its use has gradually replaced some hybrid repairs.^17^ Nevertheless, hybrid TAAA repair remains valuable in patients with complex anatomy, severe calcification, or vessel tortuosity, where endovascular stent deployment is challenging.^8^ Hybrid TAAA allows direct visceral reconstruction, intraoperative flexibility, and timely intervention in emergencies such as rupture. Moreover, prosthetic bypass grafts may achieve better long-term patency in small or diseased visceral arteries than endovascular bridging components, which can be vulnerable to restenosis or occlusion. Hybrid repair thus complements FB-EVAR depending on anatomy and clinical context.

Long-term outcomes confirmed that survival, complication rates, and aortic event-free durability were comparable between groups, reinforcing the safety of hybrid TAAA repair in patients with prior AAA open surgery. This suggests that the policy of hybrid treatment does not need to be changed in the presence of prior AAA open repair history and that consistent treatment can be provided.

In addition, exploratory IPTW analysis showed no significant differences in survival or aortic events between groups, supporting the robustness of our findings. These results suggest that, even after accounting for baseline imbalances, hybrid TAAA repair can be safely performed in patients with prior AAA open repair without increasing long-term mortality or aortic events.

### Limitations

There are some significant limitations in the present research. The sample size may not be sufficient, as this was a single-centre retrospective study. Patients in Group P had a relatively long interval (more than 10 years) from the prior AAA open repair to the visceral debranching procedure. The length of this period may have affected the degree of adhesion and influenced surgical outcomes. Different vascular surgeons performed open surgeries at various times during the study period, and 3 radiologists performed TEVAR.

## CONCLUSIONS

Although staged TEVAR may offer theoretical benefits, the risk of spinal cord injury remains, particularly under haemodynamic instability. Our findings suggest that hybrid TAAA repair can be safely performed in patients with prior AAA repair when perioperative management is optimized. Further prospective studies are warranted to confirm these results and refine patient selection.

## Supplementary Material

ivaf230_Supplementary_Data

## Data Availability

The data underlying this article will be shared on reasonable request to the corresponding author.
