# Supplementary material for: Hybrid Thoracoabdominal Aortic Aneurysm Repair After Prior Abdominal Aortic Aneurysm Repair: Safety and Outcomes
Source: Interdiscip Cardiovasc Thorac Surg. 2025 Sep 27;40(10):ivaf230. doi: 10.1093/icvts/ivaf230 (PMC12512131; doi:10.1093/icvts/ivaf230)
Supplement: ivaf230_Supplementary_Data [file ivaf230_supplementary_data.zip › Supplementary material_20250805.docx]

**Supplementary Methods:**

**Data Source**

Patient data were retrospectively extracted from electronic medical records. Hypertension, diabetes mellitus, and hyperlipidemia were defined as diagnosed by a medical doctor or use of specific medication. Renal insufficiency was defined as an estimated glomerular filtration rate of less than 60 mL/min/1.73 m2. Chronic obstructive pulmonary disease (COPD) was described as a forced expiratory volume in 1 second to forced vital capacity ratio of less than 70%. Thoracoabdominal aortic aneurysms were classified according to the Crawford classification. The number of anastomoses was defined as the total number of anastomoses, including visceral debranching and AAA repair. A spinal cord injury (SCI) was defined as damage to the spinal cord that results in a temporary or permanent loss of sensory function, motor function, or autonomic function below the level of the injury. An aortic event was defined as any of the following: aortic dissection, aortic rupture, aortic aneurysm expansion requiring surgical intervention, or aortic-related death.

**Operative management**

When the aneurysm size is greater than 60 mm, the next TEVAR is performed within a few days after the visceral debranching procedure. When the aneurysmal size is <60 mm, the next TEVAR is performed after the recovery of the activity of daily living. During AAA repair, if thoracoabdominal aneurysms >50 mm are present, a concomitant visceral debranching procedure is performed in anticipation of future hybrid TAAA repair. This is defined as preventive debranching. Operative time was measured from skin incision to skin closure. A cell-saver system was routinely used in all cases for intraoperative blood salvage.

Until 2016, cerebrospinal fluid drainage (CSF) was selectively performed based on patient risk. However, since 2017, routine preoperative CSF drainage has been avoided due to the risks associated with insertion. During TEVAR, 1000 mg of methylprednisolone was administered intravenously, and mean arterial pressure was maintained above 80 mmHg. If paraplegia occurs, CSF drainage was promptly initiated, and mean arterial pressure was raised to above 90 mmHg. TEVAR is performed under local anesthesia, allowing immediate neurologic assessment of the lower limbs. Intraoperative neurophysiological monitoring was not routinely used.

**Operative technique**

A visceral debranching procedure is performed via a median laparotomy. The abdominal aorta is usually replaced with a bifurcated graft. The quadruple graft, known as the 'InterGard Quattro' (Getinge Group Japan, Inc., Tokyo, Japan), is anastomosed to the bifurcated graft, and the visceral arteries are reconstructed using the quadruple graft. In patients who have previously undergone AAA open repair, replacement of the abdominal graft is unnecessary, and a quadrifurcated graft for visceral debranching should be anastomosed to the existing graft. TEVAR is conducted as a 2-stage procedure, consistent with our institutional protocol. Usually, TEVAR is performed under local anesthesia and sedation with intravenous anesthesia.

**Statistical analysis**

This study retrospectively analyzed all available patients (n = 67) meeting inclusion criteria. A sample size calculation, based on detecting a clinically relevant difference in 5-year overall survival (70% in Group C vs. 40% in Group P) with 80% power and a two-sided significance level of 0.05, indicated a requirement of 60 patients (40 in Group C, 20 in Group P) using a chi-square test. The slightly exceeded sample size was deemed sufficient.

Shapiro–Wilk tested normality. Parametric comparisons used Student’s or Welch’s t-test; non-parametric used Wilcoxon rank-sum. Categorical variables were compared using Fisher’s exact or χ² tests. IPTW was performed as detailed, with balance confirmed by standardized mean difference <0.2.

To explore potential treatment effects while adjusting for baseline differences between groups, an Inverse Probability of Treatment Weighting (IPTW) analysis was conducted. Propensity scores were estimated using a logistic regression model incorporating age, gender, hypertension, diabetes mellitus, chronic kidney disease, coronary artery disease, and chronic obstructive pulmonary disease. Stabilized weights were calculated and applied to the cohort to generate a pseudo-population with balanced covariates. Covariate balance was assessed using standardized mean differences, with a threshold of <0.2 considered acceptable. Weighted Cox proportional hazards models were then used to evaluate differences in overall survival and freedom from aortic events. All p values were two-tailed and considered statistically significant at P < 0.05. No multiplicity adjustments were made.

**Supplementary Figures:**

**Supplementary Figure S1. IPTW-adjusted comparison of survival probabilities after hybrid TAAA repair between patients with concomitant vs. prior open AAA repair**
Group C (n = 50) is the group in which abdominal aortic aneurysm repair was performed concomitantly, and Group P (n = 17) is the group in which abdominal aortic aneurysm repair was performed previously. After inverse probability of treatment weighting (IPTW) adjustment, the estimated overall survival at 5 years was 69% (95% CI: 57-83%) in Group C and 61% (95% CI: 39-96%) in Group P (*p* = 0.22), confirming comparable long-term survival between the groups. AAA = abdominal aortic aneurysm; CI = confidence interval; IPTW = inverse probability of treatment weighting; TAAA = thoracoabdominal aortic aneurysm.

**Supplementary Figure S2 . IPTW-adjusted comparison of freedom from aortic events after hybrid TAAA repair between patients with concomitant vs. prior open AAA repair**
Group C (n = 50) is the group in which abdominal aortic aneurysm repair was performed concomitantly, and Group P (n = 17) is the group in which abdominal aortic aneurysm repair was performed previously. After IPTW adjustment, the estimated freedom from aortic events at 5 years was 92% (95% CI: 83-100%) in Group C and 77% (95% CI: 52-100%) in Group P (*p* = 0.19), supporting comparable procedure durability between the two groups. AAA = abdominal aortic aneurysm; CI = confidence interval; IPTW = inverse probability of treatment weighting; TAAA = thoracoabdominal aortic aneurysm.

**Supplementary Table:**

**Supplementary Table 1. Graft Patency by Inflow Site (Graft Joint vs Non-Graft Joint)**

|  | **Graft Joint (n = 11)** | **Non-Graft Joint (n = 41)** | ***P* value** |
| --- | --- | --- | --- |
| Branch graft occlusion (per reconstructed branched) | 0/41 (0) | 6/ 193 (3.1) | 0.59 |

Data are presented as n (%) or median (interquartile range).
“Graft Joint” refers to the anatomical bifurcation point between the trunk and limbs of the Y-graft, adopted as the inflow site in most patients treated after 2017. “Non-Graft Joint” includes inflow from the left limb, right limb, or graft body of the Y-graft.

**Supplementary Table 2: Spinal cord injury (SCI) case summary**

| **Case** | **Group** | **Timing of TEVAR after debranching** | **Symptom** | **Timing of SCI after TEVAR** | **Cause** |
| --- | --- | --- | --- | --- | --- |
| 1 | C | Day 1665 | Paraplegia | Day 1 | Hemodynamic instability after debranching |
| 2 | C | Day 1 | Paraparesis | Day 7 | Delayed onset, recovered after CSF drainage and steroids |
| 3 | P | Day 1 | Paraplegia | Day 9 | Hypovolemic shock from postoperative ileus |
| 4 | C | Day 1 | Paraplegia | Day 0 | Sedation-induced hypotension in ICU |

**Abbreviations:** SCI = spinal cord injury; CSF = cerebrospinal fluid; ICU = intensive care unit; TEVAR = thoracic endovascular aortic repair.

**Supplementary Table 3. Details of patients who underwent visceral debranching but did not proceed to TEVAR**

| **Case** | **Group** | **Indication for debranching** | **Reason for no TEVAR** | **Outcome** |
| --- | --- | --- | --- | --- |
| 1 | C | Planned hybrid repair | Died before TEVAR in the hospital (aneurysm rupture) | Deceased |
| 2 | P | Preventive visceral debranching procedure | Died before TEVAR (aneurysm rupture) | Deceased |
| 3 | C | Preventive visceral debranching procedure | Died before TEVAR (aneurysm rupture) | Deceased |
| 4 | C | Preventive visceral debranching procedure | Awaiting | Alive |
| 5 | C | Planned hybrid repair | Died before TEVAR in the hospital (aneurysm rupture) | Deceased |
| 6 | C | Preventive visceral debranching procedure | Died before TEVAR (heart failure) | Deceased |
| 7 | C | Preventive visceral debranching procedure | Awaiting | Alive |
| 8 | C | Planned hybrid repair | Died before TEVAR in the hospital (aspiration pneumonia) | Deceased |
| 9 | C | Preventive visceral debranching procedure | Awaiting | Alive |
| 10 | C | Preventive visceral debranching procedure | Awaiting | Alive |
| 11 | C | Preventive visceral debranching procedure | Awaiting | Alive |
| 12 | C | Preventive visceral debranching procedure | Awaiting | Alive |

**Abbreviations:** AAA = abdominal aortic aneurysm; TAAA = thoracoabdominal aortic aneurysm; TEVAR = thoracic endovascular aortic repair.

**Supplementary Table 4. Summary of Aortic Events and Management**

| **Case** | **Group** | **Event Type** | **Specifics** | **Management** |
| --- | --- | --- | --- | --- |
| 1 | C | Acute dissection | Stanford type A; 84 y/o; DeBakey type II IMH, thrombosed false lumen <11 mm, aortic diameter <50 mm, hemodynamically stable | Conservative |
| 2 | C | Acute dissection | Stanford type A; 87 y/o with CKD and DM, limited surgical tolerance | Conservative |
| 3 | C | Acute dissection | Stanford type B; uncomplicated | Conservative |
| 4 | C | Arch aneurysm enlargement | Aortic arch | Open surgical repair |
| 5 | P | Stent graft infection | CT-guided drainage + antibiotics | CT-guided drainage + antibiotics |
| 6 | C | Reintervention for endoleak | Type I endoleak | Total arch replacement |
| 7 | P | Reintervention for endoleak | Type I endoleak | Additional TEVAR |
| 8 | C | Reintervention for endoleak | Type II/III endoleak | TEVAR and transarterial embolization |
| 9 | C | Aneurysm sac enlargement | Unknown endoleak | Additional TEVAR |
| 10 | C | Fatal rupture due to ascending aortic dissection | Aortic rupture | Death |

**Abbreviations:** CKD = chronic kidney disease; DM = diabetes mellitus; IMH = intramural hematoma; TEVAR = thoracic endovascular aortic repair.

**Supplementary Table 5. Covariate Balance Before and After IPTW Adjustment**

| **Variable** | **Group C Mean (Unweighted)** | **Group P Mean (Unweighted)** | **SMD (Unweighted)** | **Group C Mean (Weighted)** | **Group P Mean (Weighted)** | **SMD (Weighted)** |
| --- | --- | --- | --- | --- | --- | --- |
| Age (years) | 71.2 | 73.5 | 0.21 | 71.6 | 69.7 | 0.16 |
| Gender (% male) | 74% | 76.5% | 0.06 | 74.6% | 75.5% | 0.02 |
| HTN (%) | 90% | 88% | 0.06 | 90% | 92% | 0.06 |
| DM (%) | 18% | 12% | 0.17 | 16% | 18% | 0.05 |
| CKD (%) | 66% | 76% | 0.23 | 68% | 64% | 0.09 |
| CAD (%) | 32% | 41% | 0.19 | 34% | 31% | 0.05 |
| COPD (%) | 36% | 41% | 0.10 | 38% | 42% | 0.09 |

**Abbreviations:** IPTW = inverse probability of treatment weighting; SMD = standardized mean difference; CAD = coronary artery disease; COPD = chronic obstructive pulmonary disease; CKD = chronic kidney disease; HTN = hypertension; DM = diabetes mellitus.
