# Supplementary figures and images for: Hybrid Thoracoabdominal Aortic Aneurysm Repair After Prior Abdominal Aortic Aneurysm Repair: Safety and Outcomes
Source: Interdiscip Cardiovasc Thorac Surg. 2025 Sep 27;40(10):ivaf230. doi: 10.1093/icvts/ivaf230 (PMC12512131; doi:10.1093/icvts/ivaf230)

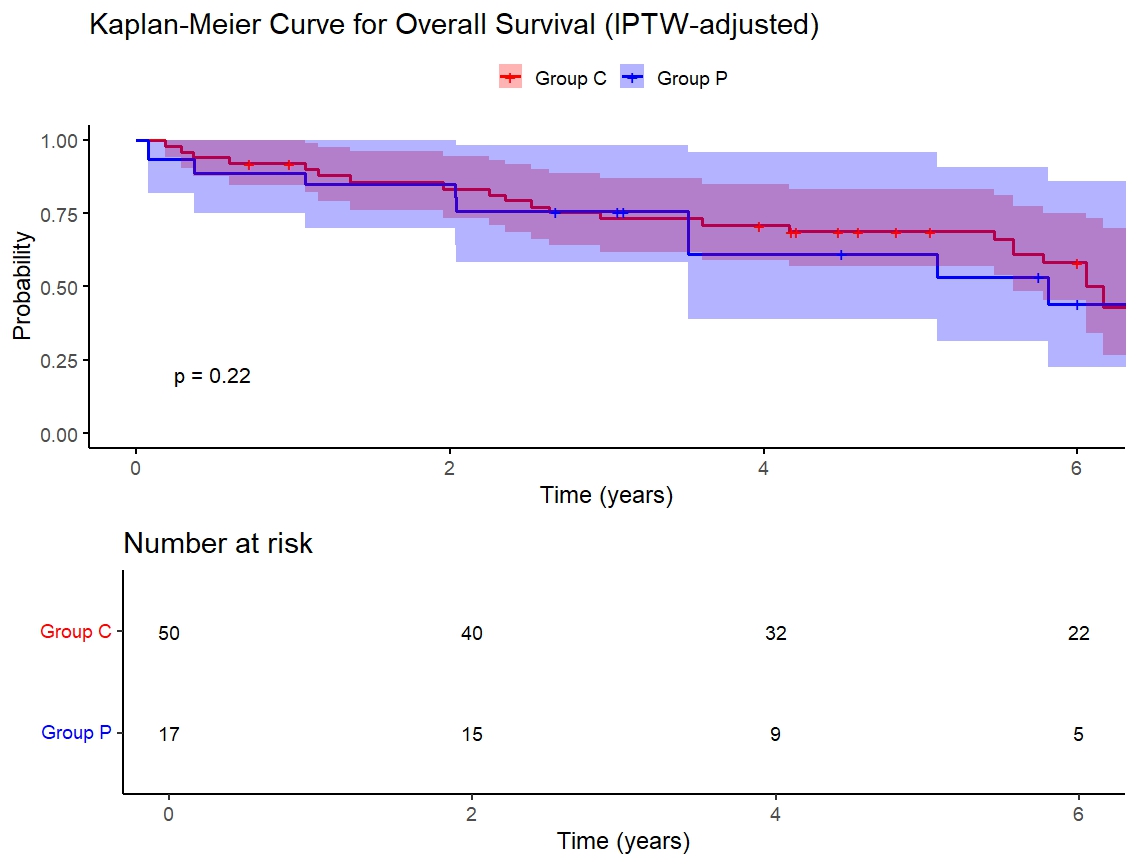

Supplement: ivaf230_Supplementary_Data [file ivaf230_supplementary_data.zip › Supplementary Figure S1.jpeg]

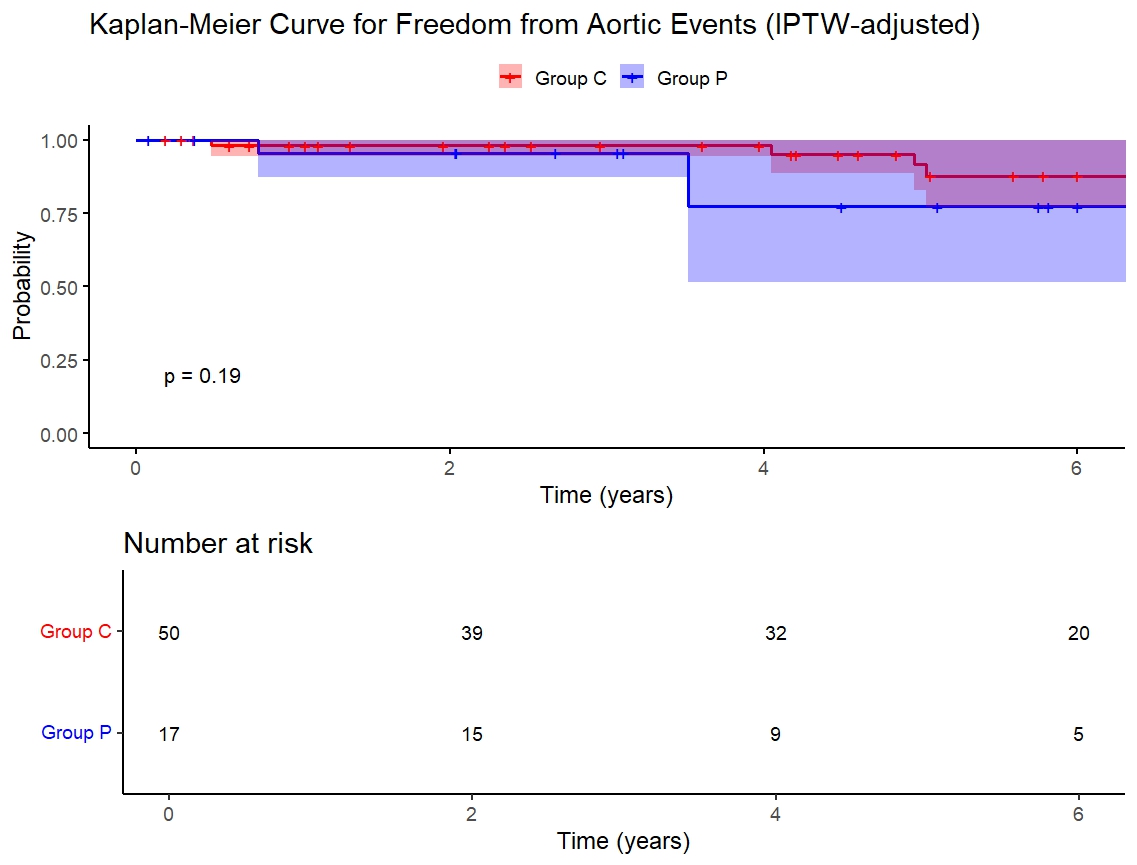

Supplement: ivaf230_Supplementary_Data [file ivaf230_supplementary_data.zip › Supplementary Figure S2.jpeg]
